# Direct and indirect influences of childhood abuse on depression symptoms in patients with major depressive disorder

**DOI:** 10.1186/s12888-015-0636-1

**Published:** 2015-10-14

**Authors:** Yumi Hayashi, Yasumasa Okamoto, Koki Takagaki, Go Okada, Shigeru Toki, Takeshi Inoue, Hajime Tanabe, Makoto Kobayakawa, Shigeto Yamawaki

**Affiliations:** Graduate School of Biomedical Sciences Programs for Biomedical Research, Hiroshima University, 1-2-3 Kasumi, Minami-ku, Hiroshima, Japan; Department of Psychiatry and Neurosciences, Institute of Biomedical and Health Sciences, Hiroshima University, 1-2-3 Kasumi, Minami-ku, Hiroshima, Japan; Department of Psychiatry, Graduate School of Medicine, Hokkaido University, Kita 15, Nishi 7, Kita-ku, Sapporo, Japan; Department of Clinical Human Sciences, Graduate School of Humanities and Social Sciences, Shizuoka University, 836 Ohya, Suruga-ku, Shizuoka City, Shizuoka Prefecture Japan

**Keywords:** Major depressive disorder, Severity of depressive symptoms, Childhood abuse, Personality, Adulthood life events, Structural equation modeling

## Abstract

**Background:**

It is known that the onset, progression, and prognosis of major depressive disorder are affected by interactions between a number of factors. This study investigated how childhood abuse, personality, and stress of life events were associated with symptoms of depression in depressed people.

**Methods:**

Patients with major depressive disorder (*N* = 113, 58 women and 55 men) completed the Beck Depression Inventory-II (BDI-II), the Neuroticism Extroversion Openness Five Factor Inventory (NEO-FFI), the Child Abuse and Trauma Scale (CATS), and the Life Experiences Survey (LES), which are self-report scales. Results were analyzed with correlation analysis and structural equation modeling (SEM), by using SPSS AMOS 21.0.

**Results:**

Childhood abuse directly predicted the severity of depression and indirectly predicted the severity of depression through the mediation of personality. Negative life change score of the LES was affected by childhood abuse, however it did not predict the severity of depression.

**Conclusions:**

This study is the first to report a relationship between childhood abuse, personality, adulthood life stresses and the severity of depression in depressed patients. Childhood abuse directly and indirectly predicted the severity of depression. These results suggest the need for clinicians to be receptive to the possibility of childhood abuse in patients suffering from depression.

SEM is a procedure used for hypothesis modeling and not for causal modeling. Therefore, the possibility of developing more appropriate models that include other variables cannot be excluded.

## Background

The onset, progression, and prognosis of major depressive disorder (MDD) are affected by many factors, including genes, environment and personality. Environmental factors related to depression are known to include maltreatment, parenting, recent life stress, and poverty. Among these environmental factors, stressful life events and childhood abuse are considered to be critical for the development of depression [1–3].

Childhood abuse is known to have long-term negative effects on mental and physical conditions, including increasing the risk for psychiatric diseases such as MDD in adulthood [2], and also affect factors related to MDD, such as growth, personality, cognitions, and behavior, as well as increasing sensitivity to life stress in childhood and adulthood [4–8]. In addition, it has been reported that the severity of MDD in adulthood is affected by childhood abuse [6, 9]. Moreover, childhood abuse is known to lead to chronic MDD, lower age of MDD onset [10], decreased responses to treatment for MDD [11, 12], and increased rate of suicide while being treated for MDD [6, 13].

It has also been suggested that personality and temperament are associated with MDD. High-Neuroticism, low-Extroversion, and low-Conscientiousness in the five-factor model of personality has been linked to the onset of MDD [14]. In particular, the association between high-Neuroticism and low-Extroversion is a robust finding in a number of studies. Moreover, high-Neuroticism might be associated with the severity of MDD [15]. Furthermore, studies using the Temperament and Character Inventory have proposed a possible association between Harm Avoidance and the onset of MDD [16]. Life stresses and the sensitization to such stresses have long been suspected of playing important roles in the onset of depression [17]. Additionally, it has been suggested that life events before the onset of depression might be related to its severity [18]. The studies discussed above have identified certain factors affecting depressive symptoms, however, few studies have indicated how these factors influence to each other.

**Fig. 1:**
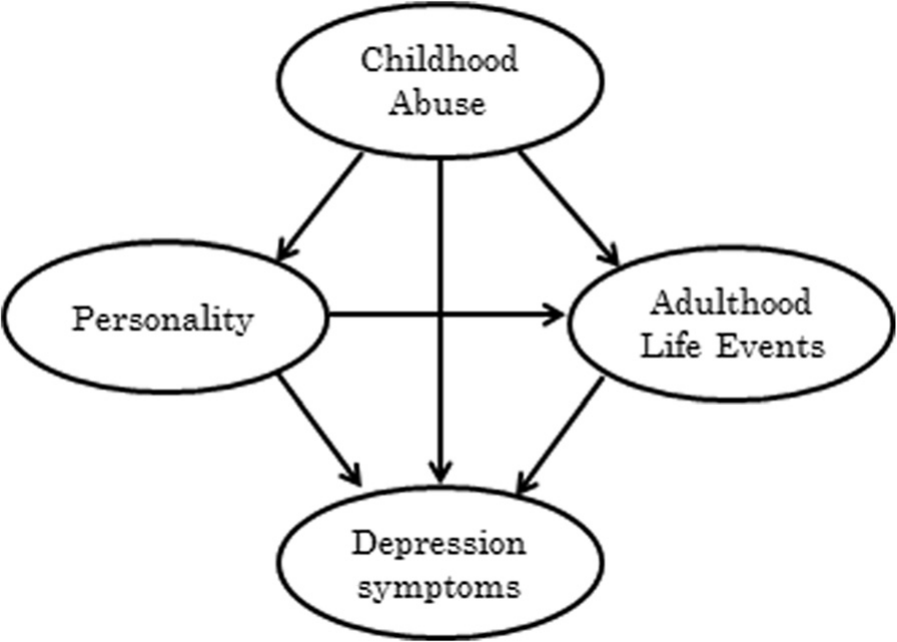
A structural equation model of the hypothesis in this study. We developed a hypothesis model that in which childhood abuse affected symptoms of depression directly and indirectly through personality and adulthood life events, and in which the personality affected stressful life events

Structural equation modeling (SEM) is considered an appropriate method for inferring the causal relationship between several variables. On the basis of previous studies, we developed the hypothetical model shown in Fig. 1. In this model, abuse in childhood, life events in adulthood, and personality are considered to have direct and indirect effects on symptoms of depression [1–6, 9, 14, 15, 17–20]. Of these, we focused on life events and personality. Instruments developed to assess these variables have high validity. We designed to include one each of internal and external risk factors affecting to depression symptom. We decided on the direction of path coefficients according to previous research and chronological order of events. In the time series, childhood abuse is considered to have occurred at the earliest. Then, childhood abuse and stressful life events in adulthood affect symptoms of depression [1–3, 6, 9, 17, 18]. Therefore, a path coefficient was proposed from childhood abuse and adulthood life events to depression symptoms. Childhood abuse also affects behaviors, character tendencies, and sensitivity to stressors in adulthood [4–8]. Therefore, a path coefficient was proposed from childhood abuse to personality and life events in adulthood. In addition, it is also known that the onset and symptoms of depression are affected by high-Neuroticism, low-Extroversion, and low-Conscientiousness [14, 15]. It has also been reported that people with a characteristically negative style of interpersonal interactions have more stressful life events [17, 19, 20]. Therefore, a path coefficient was proposed from personality to symptoms of depression and life events in adulthood.

To our knowledge, there is only one study that has analyzed the relationship between depressive symptoms, childhood abuse, character tendencies, and life events in adulthood by using SEM [21]. Nakai hypothesized that childhood abuse, stressful life events in adulthood, and affective temperament directly and indirectly affected depressive disorders, and demonstrated that childhood abuse was indirectly associated with the severity of depression, through the mediation of temperament [21]. However, their study investigated only a sample of the general population. It is known that the severity of MDD affects the response to treatment [22]. Therefore, it was considered important to evaluate how factors associated with the onset of MDD predicted the severity of depressive symptoms after the onset of the disorder. The aim of this study was to clarify how childhood abuse, personality, and stress of life events are associated with symptoms of depression in patients with depression.

## Methods

### Participants

Participants were 113 untreated, newly diagnosed MDD patients (58 women and 55 men, Mean age 41.91 years, *SD* = 11.20, Age range 25–75 years), who were patients suffering from MDD. It was difficult to locate a large number of such participants. It has been indicated that SEM requires a minimun sample of 100 [23]. Therefore, we decided to include 113 participants, so that at least 100 participants would be included in the analysis, after accounting for the possibility that certain participants would retract their consent or would give incomplete responses.

### Procedure

The ethics committees of Hiroshima University and Hiroshima University Hospital approved this study. The study was conducted between January 2012 and May 2014 in Hiroshima, Japan. The patients were recruited from six psychiatric clinics and two hospitals. All participants received an explanation from an independent clinical research coordinator (CRC), and all they signed a written informed consent form. Following this, the CRC conducted the Mini-International Neuropsychiatric Interview (MINI) with all participants. All the participants met the criteria for diagnosing a current episode of MDD according to the Fourth Edition of the Diagnostic and Statistical Manual of Mental Disorders (DSM-IV). Participants that met criteria for schizophrenia (lifetime), bipolar disorder (lifetime), eating disorder (present), substance abuse (within 6 months), and any personality disorders (present) were excluded from the study. We checked for comorbidity, including generalized anxiety disorders, panic disorders, agoraphobia, social phobia, obsessive-compulsive disorders, and posttraumatic stress disorders using the MINI. Then, participants with MDD completed the four self-report scales described below. They also gave demographic information, including age and gender, as well as information about the recurrence of the depressive disorder.

### Measurement instruments

#### Beck Depression Inventory-Second Edition (BDI-II)

The BDI-II is a self-report instrument that assesses the presence and severity of depressive symptoms [24]. It consists of 21 items that are rated on a 4-point scale ranging from 0 to 3, with higher scores indicateive of more severe symptoms of depression. It has been demonstrated that the Japanese version of the BDI-II has high internal consistency and reliability (Cronbach’s α = 0.87), and its item homogeneity has been confirmed [25].

#### Life Experiences Survey (LES)

The LES is a self-report instrument used to assess both positive and negative personal impact of recent events [26]. We used the Japanese version of the LES [27]. LES consists of 47-items that are ranked on a 7-point Lickert-type scale ranging from −3 (*extremely negative*) to +3 (*extremely positive*). Test-retest reliability of the Japanese version of LES is high with Pearson’s correlation coefficients ranging from 0.46 to 0.65 for different subscales [27]. The LES also has demonstrated validity [27].

#### Neuroticism Extroversion Openness Five Factor Inventory (NEO-FFI)

The NEO-FFI [28] is a 60-item self-report instrument used to measure the five personality domains described in the five factor model [29] --- Neuroticism, Extroversion, Openness to experience, Agreeableness, and Conscientiousness. Costa and McCrae developed this instrument in 1989. Responses to the NEO-FFI are made on a 5-point Lickert scale: ranging from 1 (*strongly disagree*) to 5 (*strongly agree*). Means and standard deviations for each personality factors have been calculated separately for men and women in a large and fairly representative sample of Japanese people. It has been demonstrated that the internal consistency (Cronbach’s α) of the Japanese version of the NEO-FFI is nearly identical to that of the original version (0.59-0.82) [30]. The validity of the Japanese version of the NEO-FFI has been confirmed [30].

#### Child Abuse and Trauma Scale (CATS)

The CATS is a self-rating questionnaire consisting of 38 items [31]. Each item is scored on a 5 point rating scale ranging between 0 (*never*) and 4 (*always*). CATS assesses the frequency of having experienced a particular abusive experience during childhood or adolescence. The scale comprises four subscales assessing subjective reports related to four aspects of childhood abuse: neglect, sexual abuse, punishment, and emotional abuse. Emotional abuse was not a part of the original scale. However, it was argued that emotional abuse was the core issue in childhood trauma and the emotional abuse subscale was developed in 1998 [32]. CATS has strong internal consistency (Cronbach’s α = 0.63-0.90) and test-retest reliability (*r* = 0 .71-0.91) [31]. The Japanese version of CATS was developed and validated by Tanabe et al. with the permission of Sanders, who originally developed the scale [33].

#### Mini-International Neuropsychiatric Interview (MINI)

The MINI is a short, structured diagnostic interview developed to screen 16 Axis I disorders and one personality disorder based on the DSM-IV [34]. The Japanese version of MINI has adequate validity [35]. Moreover, the reliability, interrater reliability, and test-retest reliability are good, or excellent, having Kappa values for major depressive disorder of over 0.75 [35]. The mean duration for administering the MINI is about 20 min.

### Model development

The hypothetical model developed in this study included the following relationships that have been identified in previous research: (1) Stressful life events and childhood abuse affect symptoms of depression [1–3, 6, 9, 17, 18]; (2) childhood abuse affects behaviors, character tendencies, and sensitivity to stressors in adulthood [4–8]; (3) the onset and symptoms of depression are affected by high-Neuroticism, low-Extroversion, and low-Conscientiousness [14, 15]; and (4) People with a characteristically negative style of interpersonal interactions report more stressful life events [17, 19, 20]. We developed the model in Fig. 1 that included two latent variables: “childhood abuse” and “personality”. Childhood abuse comprised four observed variables: neglect, punishment, sexual abuse, and emotional abuse, which were assessed by CATS subscales. “Personality” comprised three observed variables: Neuroticism, Extroversion, and Conscientiousness, which were assessed by NEO-FFI subscales. “Depression symptoms” comprised BDI-II variables, and “Adult life events” comprised variables, which were assessed by negative change score of the LES.

### Statistical analysis

Statistical analysis included (1) examining descriptive data and correlations, as well as (2) examining the model described in Fig. 1, by using SEM. We used IBM SPSS and AMOS Version 21.0 (Chicago, IL) to calculate descriptive data and correlations, and to conduct SEM. Pearson’s rank correlation coefficient was used to examine correlations between BDI-II and each subscale of CATS, NEO-FFI, and LES. We conducted Mardia’s normalized coefficient of multivariate kurtosis to examine multivariate normality.

We analyzed the maximum likelihood path model with robust standard error. We also used several fit indices for the inferential statistical evaluation of SEM, including chi-square value, Comparative Fit Index (*CFI*), and Root Mean Square Error of Approximation (*RMSEA*). Fit indices without *RMSEA* may range from 0 to 1, with lower limit of *RMSEA* being close to 0, whereas the upper limit should be less than 0.08 [36]. According to conventional criteria, an acceptable fit is indicated by *CFI* ≧ 0.95, and *RMSEA* ≦ 0.08. Moreover, a good fit is indicated by *CFI* ≧ 0.97, and *RMSEA* ≦ 0.05 [37]. However, when there are a large number of measures or constructs, it is not uncommon for the fit to be degraded [38]. We standardized and indicated all the coefficients for SEM (from −1 to 1). Indirect effects via a variable were calculated by multiplying the variables in SEM.

The level for statistically significant differences was set at *p* < 0.05.

## Results

### Background

Table 1 shows the baseline demographic and clinical characteristics of the MDD patients. It can be seen that comorbidities such as generalized anxiety disorder, panic disorder, agoraphobia, social phobia, obsessive-compulasive disorder, and posttraumatic stress disorder were observed in small numbers. Social phobia was the most common comorbidity (23/113), whereas posttraumatic stress disorder was not observed in any of the participants.

**Table 1:** Demographics characteristics of the participants

		Means ± SD or numbers (*n* = 113)
Age		41.91 ± 11.20
Gender	Male/Female	55/58
recurrence of depression	+/-	59/54
BDI-II		31.51 ± 9.19
NEO-FFI	Neuroticism	53.88 ± 7.86
	Extroversion	39.34 ± 8.77
	Openness to experience	39.95 ± 9.53
	Agreeableness	49.66 ± 10.12
	Conscientiousness	47.55 ± 9.99
CATS	Neglect	13.48 ± 10.39
	Sexual Abuse	0.57 ± 1.82
	Punishment	8.71 ± 4.23
	Emotional Abuse	5.52 ± 5.33
LES	Negative	8.19 ± 6.76
	Positive	1.25 ± 2.40
Comorbidity	Generalized anxiety disorder	3
	Panic disorder	6
	Agoraphobia	10
	Social phobia	23
	Obsessive-compulsive disorder	0
	Posttraumatic stress disorder	0

*Abbreviations*: *BDI-II* Beck Depression Inventory – 2nd edition, *NEO-FFI* Neuroticism Extroversion Openness Five Factor Inventory, *CATS* Child Abuse and Trauma Scale, *LES* Life Experiences Survey, *Negative* negative life change score, *Positive* positive life change score

### Correlation coefficients

Table 2 shows correlation coefficients between BDI-II and each subscale of NEO-FFI, CATS, and LES. Pearson’s product–moment correlation coefficients between BDI-II and subscale scores of CATS, LES, and NEO-FFI indicated significant, but weak, or moderate correlations. BDI-II had a moderate, positive correlation with Neuroticism of NEO-FFI and a weak, negative correlation with Extroversion, Agreeableness and Conscientiousness of NEO-FFI. Additionally, negative life change score of the LES were significantly related to BDI-II and the subscales of NEO-FFI and CATS. There was also a weak positive correlation between BDI-II and the negative life change score of LES. Moreover, there was a weak correlation between negative life change score of the LES and NEO-FFI subscale scores. Furthermore, neglect, sexual abuse, and emotional abuse subscales of CATS were significantly correlated with LES.

**Table 2:** Correlation coefficients between BDI-II and each subscale of NEO-FFI, CATS, and LES

	BDI-II	NEO-N	NEO-E	NEO-O	NEO-A	NEO-C	CATS-N	CATS-S	CATS-P	CATS-E	LES-N	LES-P
BDI-II												
NEO-N	.47**											
NEO-E	-.25**	-.31**										
NEO-O	-.05	-.03	.07									
NEO-A	-.19*	-.30**	.12	.15								
NEO-C	-.30**	-.35**	.28**	.01	.09							
CATS-N	.28**	.28**	-.06	.13	-.16	-.10						
CATS-S	.15	.15	.00	-.00	-.06	-.05	.45**					
CATS-P	.29**	.01	-.03	-.02	-.14	.03	.42**	.17				
CATS-E	.39**	.27*	-.04	.18	-.20*	-.16	.74**	.38**	.48**			
LES-N	.29**	.24*	-.07	.03	-.17	-.14	.31**	.33**	.07	.25**		
LES-P	.03	.06	.00	.25**	-.05	.01	.27**	.50**	.07	.17	.18	

**p < .01, *p < .05

*Abbreviations*: *BDI-II* Beck Depression Inventory – 2nd edition, *NEO-N* Neuroticism of Neuroticism Extroversion Openness Five Factor Inventory, *NEO-E* Extroversion of Neuroticism Extroversion Openness Five Factor Inventory, *NEO-O* Openness to experience of Neuroticism Extroversion Openness Five Factor Inventory, *NEO-A* Agreeableness of Neuroticism Extroversion Openness Five Factor Inventory, *NEO-C* Conscientiousness of Neuroticism Extroversion Openness Five Factor Inventory, *CATS-N* Neglect of Child Abuse and Trauma Scale, *CATS-S* Sexual Abuse of Child Abuse and Trauma Scale, *CATS-P* Punishment of Child Abuse and Trauma Scale, *CATS-E* Emotional Abuse of Child Abuse and Trauma Scale, *LES-N* Negative life change score of Life Experiences Survey, *LES-P* Positive life change score of Life Experiences Survey

### Structural equation modeling

**Fig. 2:**
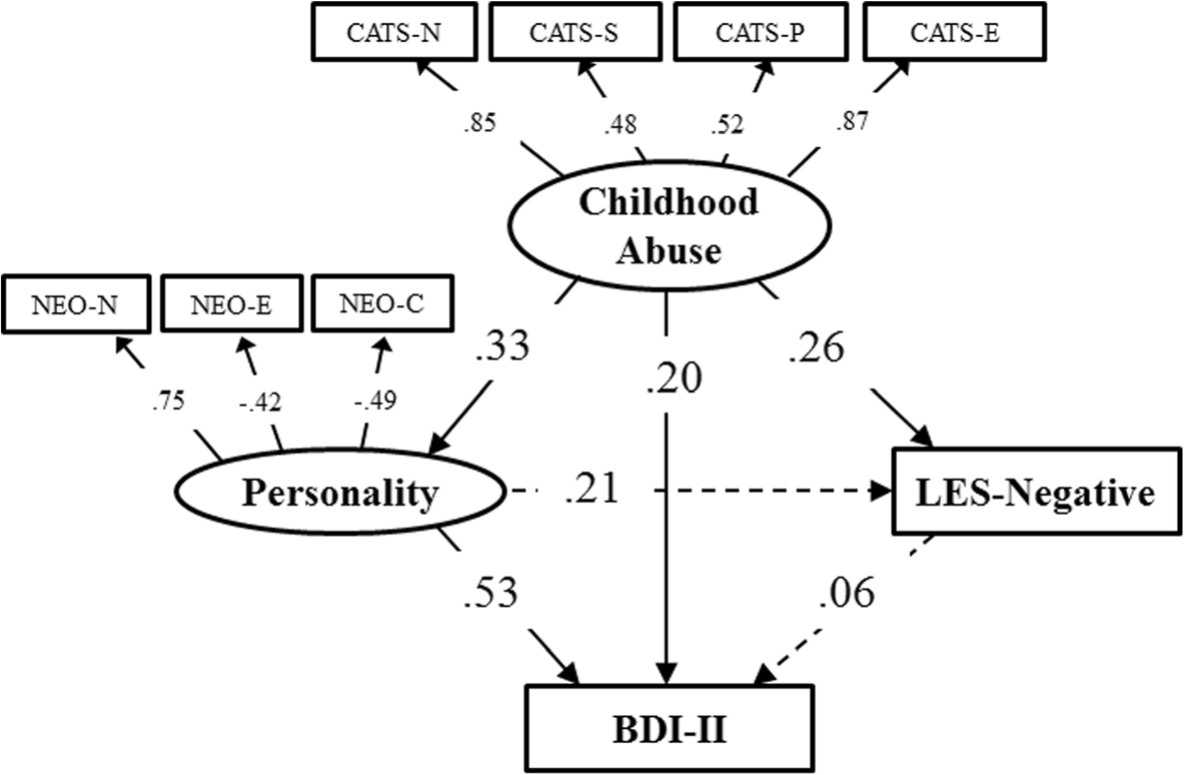
Results of covariance structure analysis of structural equation model (SEM). These are the results of covaliance structure analysis in the SEM with childhood abuse (CATS), personality (N, E, C of NEO-FFI), adulthood life events (Negative score of LES), and depression symptoms (BDI-II) for 113 patients of major depressive disorder. The allows with continuous lines represent the statistically significant paths, and the dashed lines show the non-significant paths. The numbers on the allows show the standardized path coefficients (minimun −1, maximum 1). Indirect effects indicate the effect mediated by the other valiables. There is direct influence from childhood abuse to severity of depression. There is also indirect effect via personality. Chi-square value: 28.83 (p=.19); CFI=.98, RMSEA=.05: a good fit.indirect effect= .3749, square sums of multiple correlation coefficient=.43. continuous line: p<.05, dashed line: p=not significant. *Abbreviations*: *BDI-II* Beck Depression Inventory – 2nd edition, *NEO-N* Neuroticism of Neuroticism Extroversion Openness Five Factor Inventory, *NEO-E* Extroversion of Neuroticism Extroversion Openness Five Factor Inventory, *NEO-C* Conscientiousness of Neuroticism Extroversion Openness Five Factor Inventory, *CATS-N* Neglect of Child Abuse and Trauma Scale, *CATS-S* Sexual Abuse of Child Abuse and Trauma Scale, *CATS-P* Punishment of Child Abuse and Trauma Scale, *CATS-E* Emotional Abuse of Child Abuse and Trauma Scale, *LES-Negative* Negative life change score of Life Experiences Survey, *CFI* Comparative Fit Index, *RMSEA* Root Mean Square Error of Approximation

Figure 2 shows results of covariance structure analysis of SEM. An overall SEM of the theoretical relationships shown in Fig. 1 was conducted by using all the data. We conducted Mardia’s normalized coefficient of multivariate kurtosis to examine multivariate normality. The results indicated that multivariate normality was not observed in this model. It has been suggested that maximum likelihood estimation with robust standard error should be conducted when multivariate normality is not observed [39]. Therefore, we conducted the path model with this analysis. The results indicated that the model fit was adequate (χ^2^ (23) = 28.83, *p* =0.19, *CFI* = 0.98, *RMSEA* = 0.05; Fig. 2). This indicated that the path coefficient from Personality to Depression Symptoms was strong, whereas other path coefficients were weak, and certain path coefficients were not significant.

The patients had a significant, positive path correlation between Childhood Abuse and BDI-II scores, and with the negative life change score of the LES. Moreover, the negative life change score of the LES was not significantly correlated with Personality; whereas it was significantly correlated with Childhood Abuse. There was also a positive and significant path coefficient from Childhood Abuse to Personality, as well as from Personality to BDI-II. However, there was no significant path coefficient from the negative life change score of the LES to BDI-II.

## Discussion

The purpose of this present study was to identify how childhood abuse, personality, and stress of life events predicted symptoms of depression. We used SEM and evaluated and compared the effects of multiple factors on the severity of depression in MDD patients. Results indicated three important findings. Firstly, childhood abuse directly predicted the severity of depression. Secondly, childhood abuse predicted the severity of depression indirectly through the mediation of personality. Thirdly, the negative life change score of the LES was affected by childhood abuse; however, the negative life change score of the LES did not predict the severity of depression. This is the first report of a relationship between multiple factors, including childhood abuse, personality, and stress of life events, and the severity of depression in MDD patients.

The hypothesized model of this study was analyzed by using SEM. Results indicated that the model had good fit indices. Nakai also examined a similar model in the general population by using SEM, and reported similar fit indices and similar results to this study [21]. Although there were certain differences in temperament and personality of the participants, between this and Nakai’s study, nevertheless it supported the validity of current findings.

Results of the current study indicated that childhood abuse directly predicted the severity of depression. Correlation analysis also indicated that MDD patients showed a weak, but a positive correlation between neglect, punishment, and emotional abuse subscales of CATS and BDI-II. Previous studies have indicated that abuse in childhood was directly and indirectly associated with the severity of depression symptoms in adulthood [3, 9, 10]. These previous findings were rainforced by current findings. Kessler and Magee suggested that childhood adversity could first lead to the onset of depression in children and adolescents, and that depression in childhood and adolescence affected depression in adulthood [3]. Korkeila reported that the interaction between childhood abuse and life stress in adulthood increased depression in adulthood, because abuse affected coping styles, the attachment style, and resilience, and because depression impaired interpersonal relationships [9]. Factors associated with childhood abuse and/or the severity of depression leading to the onset of depression, such as precipitating factors such as family history [40, 41], genes of specific neurotransmitter receptors [42], mental vulnerability [5], negative cognitive style [43]; as well as moderators, such as self-esteem, coping style, and social support [44] are considered precipitating factors of depression. In addition to these the mechanisms of the onset of depression might also be associated with the severity of depression. It is suggested that future studies should investigate the relationships between different mechanisms of the severity of depression.

Childhood abuse could cause changes in the development of personality, and personality could be a predictor of the severity of depression. The results of this study corroborated the relational model in which abuse could change the personality reported in a previous study [4]. Petersen indicated a trend for a positive relationship between the personality and the severity of depression [15]. To our knowledge, this study is the first to indicate a significant positive relationship between personality and the severity of depression. However, it is unclear whether personality is best conceptualized as a predisposition for depression, or if the severity of depression increases the likelihood of expressing personality changes [15]. We were unable to evaluate these competing possibilities, because data on premorbid personality levels were not investigated in this study.

Results of the study also indicated a positive effect from abuse to life stress events in MDD patients. It has been reported that childhood abuse is related to negative cognitive styles, and a lower threshold for life stress events [5, 9]. The results also supported these previous findings. Life events are considered to be major factors that are associated with the onset and severity of depression [18, 45]. The correlation analysis of this study indicated a moderate, but a significantly positive relationship between negative life change scores of the LES and the severity of depression. SEM, however, did not indicate that negative life change score of the LES predicted the severity of depression. There could be several reasons for this lack of predictive power: abuse, personality, and other factors might have caused spurious correlations between negative life change score of the LES and the severity of depression. LES is a scale assessing feelings about life events and not the actual events themselves. The evaluation and the style of cognizing life events are known to affect symptoms of depression [46]. As a result, Type 2 errors could have occurred in path coefficients, because of the small sample size. The criterion for judging life event is affected by abuse, and SEM did not indicate that LES predicted the severity of depression. Moreover, chronic stress could have made a stronger contribution to the severity of depression than acute stress [47]. Furthermore, LES is a scale evaluating life events happened in the previous year. Also, LES dose not evaluate chronic stress. The factors discussed above could have accounted for the inability of negative life events to predict the severity of depression.

The results of this study should be considered in light of following limitations. Firstly, there is the possibility of age and memory biases related to childhood trauma [3, 5, 48]. In this study, participants aged between 25 and 75 years were recruited. Previous studies have reported that childhood abuse affected adolescents and young adults more strongly than middle age and elder people [3, 5, 48]. However, other studies have reported that childhood abuse has certain effects on elderly people [48]. The possibility of memory biases related to childhood abuse has also been focused [3, 5]. Moreover, the possible generation effect related to the age bias has been indicated [48]. Therefore, it is suggested that further studies recruiting a larger sample of MDD patients are needed to investigate the generation effects of the age bias. Secondly, SEM is a hypothesis modeling technique and not a technique or causal modeling. Therefore, it is possible that there are more appropriate models that include other variables. Thirdly, the effect of recall bias cannot be excluded in this study. Bernet and Stein suggested that depressed patients are more likely to recall abusive childhood experiences than healthy controls [10]. Therefore, prospective cohort studies starting from childhood are needed to exclude the effects of recall bias in depressed patients. Fourthly, the sample size in this study was small, and as a result, Type 2 error could have occurred in the path coefficient.

In summary our results suggested that childhood abuse affects directly and indirectly severity of depression in adulthood. Childhood abuse predicted the severity of depression, and the indirect effect through personality. It is known that depressed patients that have experienced childhood abuse react poorly to antidepressant medications [11]. It has also been suggested that, combined antidepressant and psychotherapy is more effective for such patients than either therapy alone [12]. Moreover, childhood abuse is known to increase the suicide rate [6, 13]. These results suggest the need for psychotherapeutic interactions for patients that have experienced childhood abuse, if they also have severe depression. It is suggested that in the future, prospective studies with larger cohorts should be used to investigate the underlying mechanisms of findings of this study.

## Conclusions

Our results suggested that childhood abuse directly and indirectly increased the severity of depression. The indirect effect was through personality, rather than through recently life events. It is known that depressed patients that have experienced childhood abuse react poorly to antidepressant medications [11]. It has also been suggested that, combined antidepressant and psychotherapy is more effective for such patients than either therapy alone [12]. Moreover, childhood abuse is known to increase the suicide rate [6, 13]. These results suggest the need for psychotherapeutic interactions for patients that have experienced childhood abuse, if they also have severe depression. Prospective studies with larger cohorts should be used to investigate the underlying mechanisms of findings of this study.

## Abbreviations

MDD: Major depressive disorder
SEM: Structural equation modeling
CRC: Clinical research coordinator
LES: Life Experiences Survey
BDI-II: Beck Depression Inventory-II
NEO-FFI: Neuroticism Extroversion Openness Five Factor Inventory
CATS: Child Abuse and Trauma Scale
CFI: Comparative fit index
RMSEA: Root Mean Square Error of Approximation
